# Optimal Dose of Serotonin Reuptake Inhibitors for Obsessive-Compulsive Disorder in Adults: A Systematic Review and Dose–Response Meta-Analysis

**DOI:** 10.3389/fpsyt.2021.717999

**Published:** 2021-09-23

**Authors:** Jiao Xu, Qinjian Hao, Ruiyi Qian, Xingyu Mu, Minhan Dai, Yulu Wu, Yiguo Tang, Min Xie, Qiang Wang

**Affiliations:** ^1^Department of General Practice, West China Hospital of Sichuan University, Chengdu, China; ^2^The Center of Gerontology and Geriatrics, West China Hospital of Sichuan University, Chengdu, China; ^3^West China School of Medicine, Sichuan University, Chengdu, China; ^4^Mental Health Center and Psychiatric Laboratory, State Key Laboratory of Biotherapy, West China Hospital of Sichuan University, Chengdu, China; ^5^West China Brain Research Center, West China Hospital of Sichuan University, Chengdu, China

**Keywords:** obsessive-compulsive disorder, serotonin reuptake inhibitors, systematic review, meta-analysis, optimal dose

## Abstract

**Background:** Obsessive-compulsive disorder (OCD) is a common chronic mental disorder with a high disability rate. Serotonin reuptake inhibitors (SRIs), including selective serotonin reuptake inhibitors (SSRIs) and tricyclic antidepressants, such as clomipramine, are the most common choices for the pharmacological treatment of OCD. Optimizing their use is pivotal in guiding clinical practice of OCD. However, there are few studies on the optimal dose of SRIs and there is controversy about their dose–response relationship and optimal target dose. Therefore, the objective of this study was to summarize the relationship between the dose and effect of SRIs, as well as the optimal dose of SRIs for OCD, as to propose future research directions.

**Methods:** Medline, Embase, Biosis, PsycINFO, Cochrane Central Register of Controlled Trials (CENTRAL), Web of Science, and CINAHL were searched for relevant publications, and the search was up to February 22, 2020. We used a one-stage, robust error meta-regression (REMR) model to deal with the correlated dose–response data for SRIs from different studies. Doses of SRIs were converted to fluoxetine equivalents when performing dose–response analysis. Review Manager Program Version 5.3 and STATA software package (version 15.1) were applied to analyze data. The study protocol was registered with PROSPERO (number CRD42020168344).

**Results:** Eleven studies involving 2,322 participants were included in final analysis. For SRIs, the dose–efficacy curve showed a gradual increase trend in the 0–40-mg dose range and then had a decreased trend in doses up to 100 mg fluoxetine equivalent. Dropouts due to adverse effects gradually increased throughout the inspected dose slope. The curve of dose of all-cause dropouts suggested no relationship between them. Sensitivity analysis proved that these results were robust.

**Conclusion:** The systematic review found that the optimal dose for efficacy was about 40mg fluoxetine equivalent. Tolerability decreased with increased doses, and there was no significant correlation between acceptability and doses of SRIs. Therefore, the optimal dose of SRIs needs to consider effectiveness and tolerability.

**Systematic Review Registration:** [PROSPERO], identifier [CRD42020168344].

## Introduction

Obsessive-compulsive disorder (OCD) is characterized by obsessions (recurrent, intrusive thoughts, images, or impulses) and/or compulsions (behaviors or mental actions taken repeatedly to decrease anxiety) (1). OCD is one of the 10 most debilitating physical and mental disorders (2) with a lifelong prevalence of 2–3% in the general population (3–6). Researchers have made efforts for several decades to improve the identification of effective treatments, including psychotherapy, pharmacotherapy, and combined treatments. Based on the data of clinical trials, cognitive-behavioral therapy (CBT) and serotonin reuptake inhibitors (SRIs) were recommended as mainstream treatments for OCD for both safety and effectiveness (7). In terms of pharmacotherapy, a comprehensive meta-analysis (8) indicated that SRIs, such as tricyclic antidepressants, clomipramine, and some selective serotonin reuptake inhibitors (SSRIs), were highly efficacious for OCD. Later, a Cochrane review (9) corroborated the efficacy of all SSRIs (including citalopram, fluoxetine, fluvoxamine, paroxetine, and sertraline), with no reliable differences. Clomipramine was consistently proven to be as effective or as even slightly better than SSRIs in the treatment of OCD, despite its less favorable side effects (10–13). Previous evidence revealed that clomipramine had anticholinergic side effects, such as dry mouth, blurred vision, constipation, fatigue, tremor, hyperhidrosis, and an increased risk of arrhythmias and seizures with daily doses >200 mg, which were rarely seen in SSRI therapy (14). Nevertheless, a recent review suggested that SSRIs (specifically fluoxetine) were the antidepressants most associated with manic/hypomanic episodes across the entire subjects, but no significant differences were found in clomipramine (15).

However, there were still some controversies over the dose dependency and optimal target dose of SRIs. Higher and rapidly increased SRI doses were recommended by many OCD experts when treating OCD compared with other conditions, such as anxiety disorders and major depressive disorder (14, 16). Similarly, American Psychiatric Association Practice Guidelines recommended a higher target dose for OCD than depression (7). Moreover, patients who failed to respond positively to multiple SSRIs at the maximum tolerated dose for a sufficient duration (at least 2 months) were diagnosed with treatment-resistant refractory OCD (16). Therefore, compared with many other mental disorders, patients with OCD were treated with SSRIs at higher doses before receiving replacement or augmentation therapies (17). Nevertheless, controlled trials have not reached consistent conclusions as to whether higher doses of SSRIs can lead to beneficial therapeutic effects, since they may carry a higher burden of side effects for patients at the same time. Some fixed-dose studies showed that higher doses of SRIs could provide better treatment efficacy (18–21), while some did not (22, 23). Bloch et al. (17) reported that higher doses of SSRIs were more effective when treating adults with OCD and were associated with a significant increase in the proportion of people who dropped out due to side effects, but the dose of SSRI was independent of the total number of all-cause dropout. Studies on a fixed dose of clomipramine for OCD treatment were rare, and the dose–response relationship was unclear.

To sum up, available reviews about the dose dependency of SRIs were scarce and the evidence was inconsistent. Given this, we conducted a dose–response meta-analysis of fixed-dose studies of commonly used antidepressants, including clomipramine and all of the SSRIs for the treatment of adults diagnosed with OCD, to further define the dose–response relationship of SRIs and give more insights into the optimal dose of SRIs for OCD.

## Methods

### Search Strategy

The meta-analysis was conducted based on the Preferred Reporting Items for Systematic Reviews and Meta-Analyses (PRISMA) statement (24). The study protocol was registered with PROSPERO (number CRD42020168344). MEDLINE, Embase, Biosis, PsycINFO, Cochrane Central Register of Controlled Trials (CENTRAL), Web of Science, and CINAHL were searched without restrictions of language from database inception to February 22, 2020. Search strategies of the study combined terms “obsessive compulsive disorder” and “citalopram or escitalopram or fluoxetine or fluvoxamine or paroxetine or sertraline or clomipramine” (Appendix 1). In addition, we also manually screened the relevant reviews in the reference list to find additional studies.

### Inclusion and Exclusion Criteria

Single- or double-blind, randomized controlled trials (RCTs) were included to compare antidepressants among themselves or with placebo as oral monotherapy for the acute-phase treatment of adults (aged 18 years or older), with an initial diagnosis of OCD according to standard operationalized diagnostic criteria. We excluded trials of antidepressants for patients with OCD and severe concomitant physical conditions. Studies of patients with treatment resistance, concomitant serious medical illnesses, and relapse-prevention studies were also excluded. The study focused on the most commonly used antidepressants, mainly SRIs such as clomipramine, and SSRIs.

### Data Extraction

Two reviewers (JX and QH) screened the search results and retrieved full-text articles independently. In case of doubt, a third reviewer (QW) participated. Three reviewers (JX, QH, and MX) performed data extraction independently, using a standard data extraction form in Microsoft Excel 2010. This included verifying study eligibility, sample size, age (mean, SD, and range), average duration of OCD (mean and SD), gender, comorbidity, diagnostic criteria, treatment time, active agent and dose, outcomes (primary and secondary measures), reported statistics, length of follow-up, and number of participants lost and excluded at each stage of the trial. For conflicting data entries, reviewers performed algorithm checks. Differences were discussed, and if no consensus was reached, we turned to another examiner (QW). If the information was missing or unclear, the study authors were contacted.

### Risk of Bias Across Studies

We used Cochrane Collaboration's risk-of-bias tool to insert figures to independently assess the risk of bias in the main results of RCTs (25) and evaluated the risk of bias in allocation sequence generation, allocation concealment, blinding of researchers and participants, blinding of result evaluators, selective outcome reporting, and other bias. If none of these areas were rated as high risk of bias and no more than three areas were rated as unclear risk, then this study was classified as low risk of bias; if one area was rated as high risk of bias or no one was rated as high risk of bias but more than three areas were rated as unclear risk, then this study was rated as moderate risk of bias; and all other cases were considered to have high risk of bias (26, 27). We conducted funnel plots to supervise the reporting bias (Appendix 2). If the funnel plot was symmetric, there may be no bias (28).

### Test of Heterogeneity

We performed statistical analysis by Review Manager Program Version 5.3 and STATA version 15, and statistical significance was set at a two-tailed p < 0.05. Heterogeneity was tested by Q test among studies and was evaluated by *p*-value and *I*^2^ value. When *p* > 0.1 and *I*^2^ ≤ 50%, it is considered that there is no obvious heterogeneity between the studies, and the fixed-effect model is selected; when *p* < 0.1 and *I*^2^ ≤ 50%, the heterogeneity is acceptable, and the fixed effect is the selected model; and *p* < 0.1 with *I*^2^ > 50% suggested that there is obvious heterogeneity between the studies. It is necessary to analyze the causes of the heterogeneity, carry out sensitivity analysis, and then select the random effects model. Publication bias was evaluated by means of the Egger's test (Appendix 2).

### Outcomes

The following outcomes were included after 10 weeks of treatment (range 8–13 weeks): (1) the primary outcome is the mean difference measured by the Yale-Brown Obsessive-Compulsive Scale (Y-BOCS). (2) The secondary outcomes are dropouts due to all causes, which was interpreted as an overall indicator of treatment acceptability, and discontinuations due to adverse effects, as an indicator of treatment tolerability.

### Dose Conversion Across Drugs

Dose equivalent can be calculated in different ways (29). One method using flexible doses in double-blind studies assumed the optimum doses to be equivalent (30). In this review, we adopted the method of Hayasaka and colleagues for the main analysis (31). Previous studies of SRIs' dose dependence used similar conversion algorithms (32, 33). In the absence of empirical data on dose conversion, we presumed that the daily defined dose (34) was equal. The dose conversion algorithms are shown in Table 1.

**Table 1:** Antidepressant dose equivalence (mg) according to previous studies.

	**Bollini et al. (32)**	**Hayasaka et al. (31)**	**Jakubovski et al. (33)**	**WHO (34)**
Citalopram	30	NR	33.3	20
Clomipramine	100	58	NR	100
Escitalopram	NR	9	16.7	10
Fluoxetine	20	20	20	20
Fluvoxamine	100	72	100	100
Paroxetine	20	17	20	20
Sertraline	83	49.3	120	50

*These doses were calculated based on fluoxetine equivalents of serotonin reuptake inhibitors (SRIs) used in previous meta-analytic studies of antidepressants and according to the American Psychiatric Association dose recommendations for individual SRIs in obsessive–compulsive disorder (OCD). NR, none reported*.

### Data Analyses

Studies of all SRIs were synthesized to estimate the dose dependence of the three main outcomes. We used the method of Hayasaka and colleagues to convert the doses to fluoxetine equivalents (31), with the daily defined dose method (34) as supplementary. In this analysis, we utilized a one-stage, robust error meta-regression (REMR) model to handle the synthesis of relevant dose–response data from different studies (35). This was done by setting three fixed knots at the 5, 50, and 95 quartiles or setting three random knots on the quartiles of the dose distribution) (36). The one-stage REMR approach was executed in STATA software package (version 15.1). Such approach estimates the association between the dose and mean difference (MD) for the primary outcome, the dose and the risk ratio (RR) for the second outcomes, side-effect-related dropouts, and all-cause dropouts, considering that the heterogeneity, within and across studies, was applied simultaneously in a single model.

The following sensitivity analyses were performed to test the robustness of the major findings: (1) set different doses and numbers of knots and (2) apply the latest daily defined dose conversion algorithm (34) (Appendix 2).

### Role of the Funding Source

The sponsor did not participate in the study design, data collection, data analysis, data interpretation, or writing of this manuscript. The corresponding author had full access to all data in this study and was ultimately responsible for the decision to submit for publication.

## Results

### Study Selection

**Figure 1:**
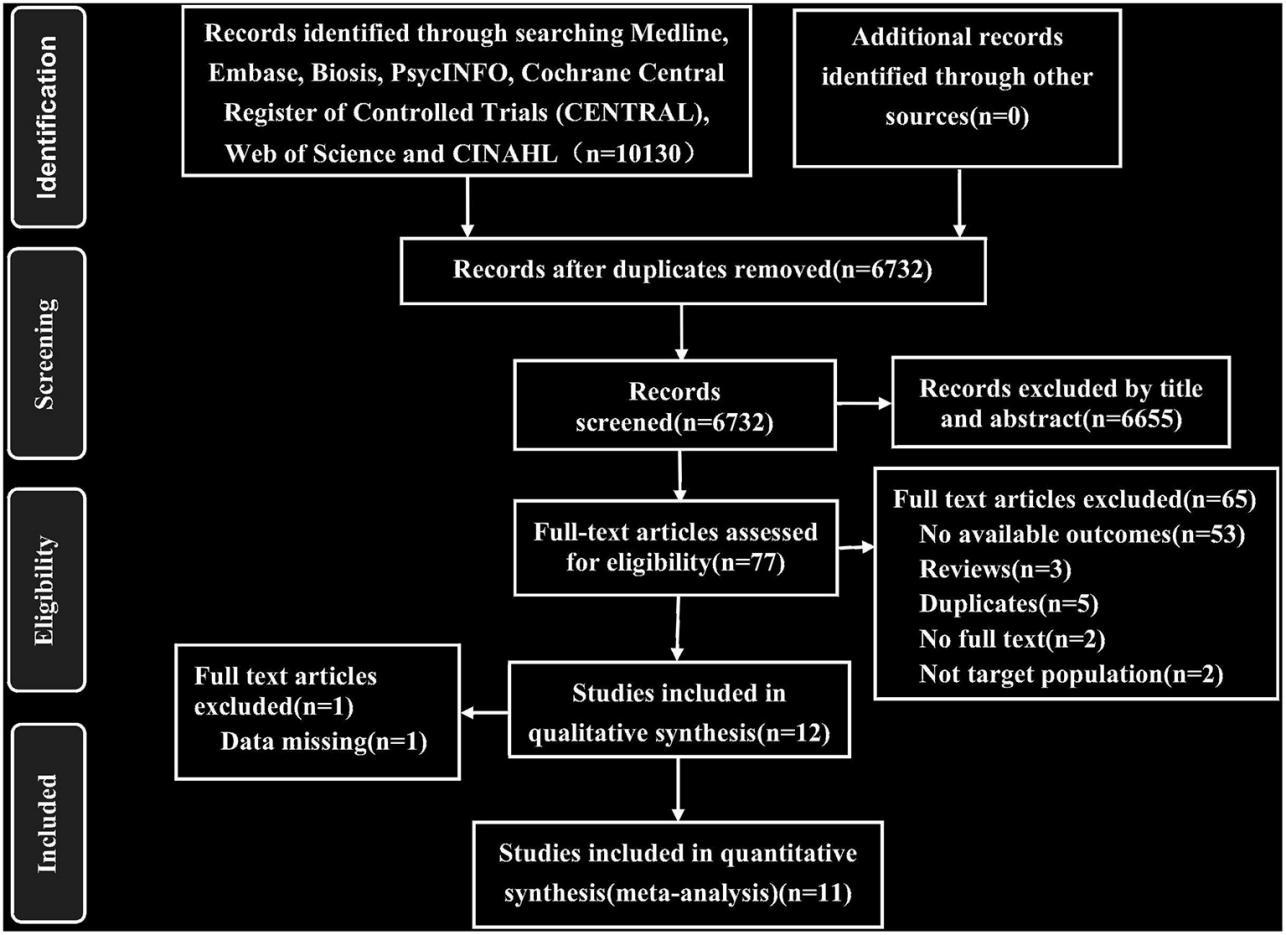
The flowchart of study selection.

As described in Figure 1, we identified 10,130 published records through automatic search, manual search, and contact with authors and retrieved 77 full-text articles after excluding 6,655 reports based on titles and abstracts. We screened these articles and eventually included 11 studies with 2,322 participants. The inter-rater agreement was evaluated in the two stages of screening and full-text review, and Cohen's κ were 0.84 and 0.95, respectively. There was no language restriction in the retrieval process. Among the 11 articles, there were one Japanese and 10 English articles. The heterogeneity and sensitivity analysis of the Japanese article showed no difference. The 11 studies included 35 treatment groups: eight for placebo, three for citalopram, two for escitalopram, seven for fluoxetine, four for paroxetine, six for sertraline, and two for clomipramine. Six of the studies had four treatment groups, one had three treatment groups, and four had two treatment groups. The median length of the trials was 10 weeks (ranging from 8 to 13 weeks). The characteristics of the sample were as follows: (1) the mean age was 37.13 years (SD 3.68), and (2) 1,142 (49.1%) of 2,322 participants were women. See Appendix 3 for the characteristics of the included studies.

### Risk of Bias Within Studies

**Figure 2:**
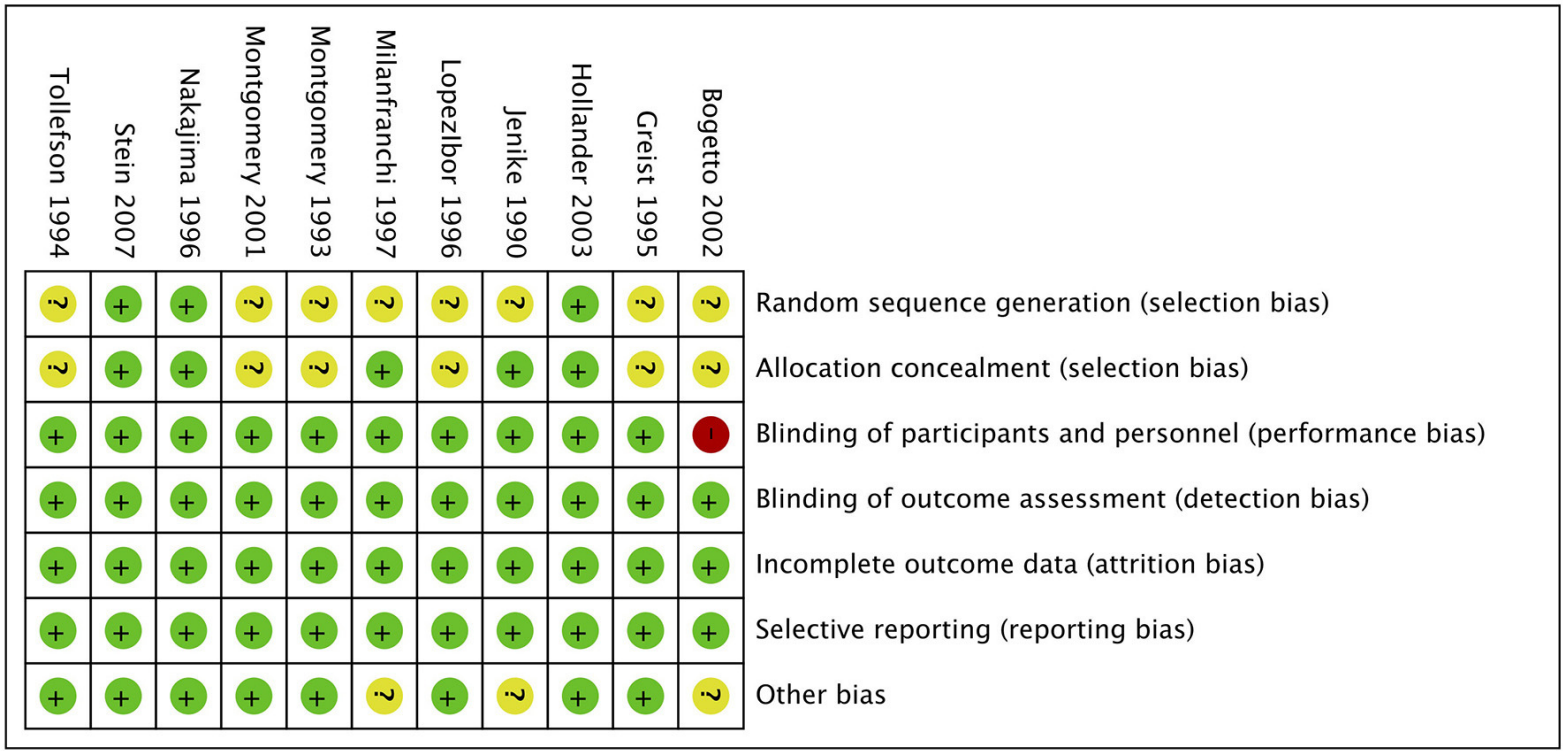
Summary of risk bias in clinical controlled trials of SRIs in OCD adults. Green circles, low risk of bias; yellow circles, unclear risk of bias; red circles, high risk of bias.

**Figure 3:**
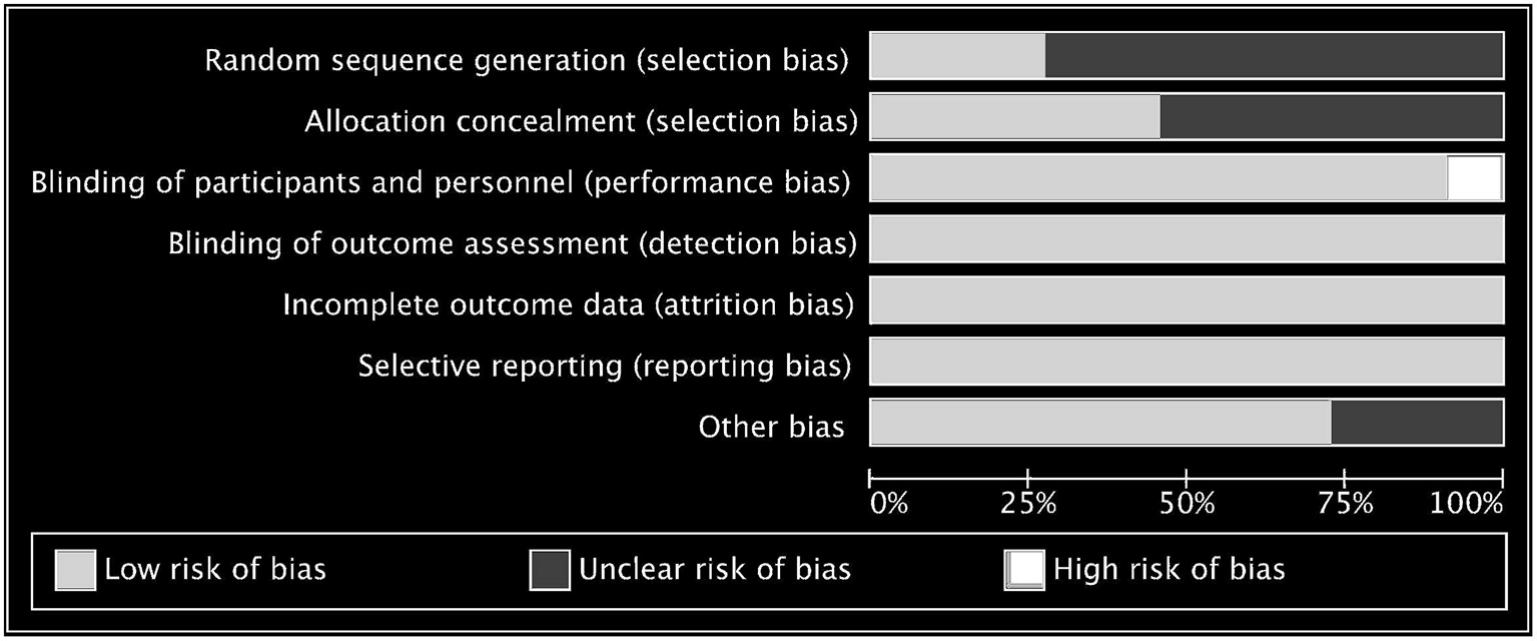
The risk of bias assessment for the individual domains.

The risk of bias of the 11 included studies are shown in Figure 2, and the risk-of-bias assessment for the individual domains is shown in Figure 3. We excluded studies with high risk of bias. Generally, the methods of random sequence generation and allocation concealment were not depicted in detail, so they were encoded as unclear. Some studies with very small sample sizes which were not sure about other biases were also coded as unclear. The percentages of the individual domains with high, unclear, and low risks of bias in the risk assessment were as follows: 0, 72.7, and 27.3% for randomization, 0, 54.5, and 45.5% for allocation concealment, 9.1, 0, and 90.9% for blinding toward patients and researchers, 0, 0, and 100% for masking of outcome assessment, 0, 0, and 100% for incomplete outcomes, 0, 0, and 100% for selective reporting, and 0, 27.3, and 72.7% for other biases. The results of the overall bias risk rating were as follows: 10 studies (91%) had low risks of bias, 1 study (9%) had a medium risk of bias, and no study had a high risk of bias.

### Synthesis of Results

**Figure 4:**
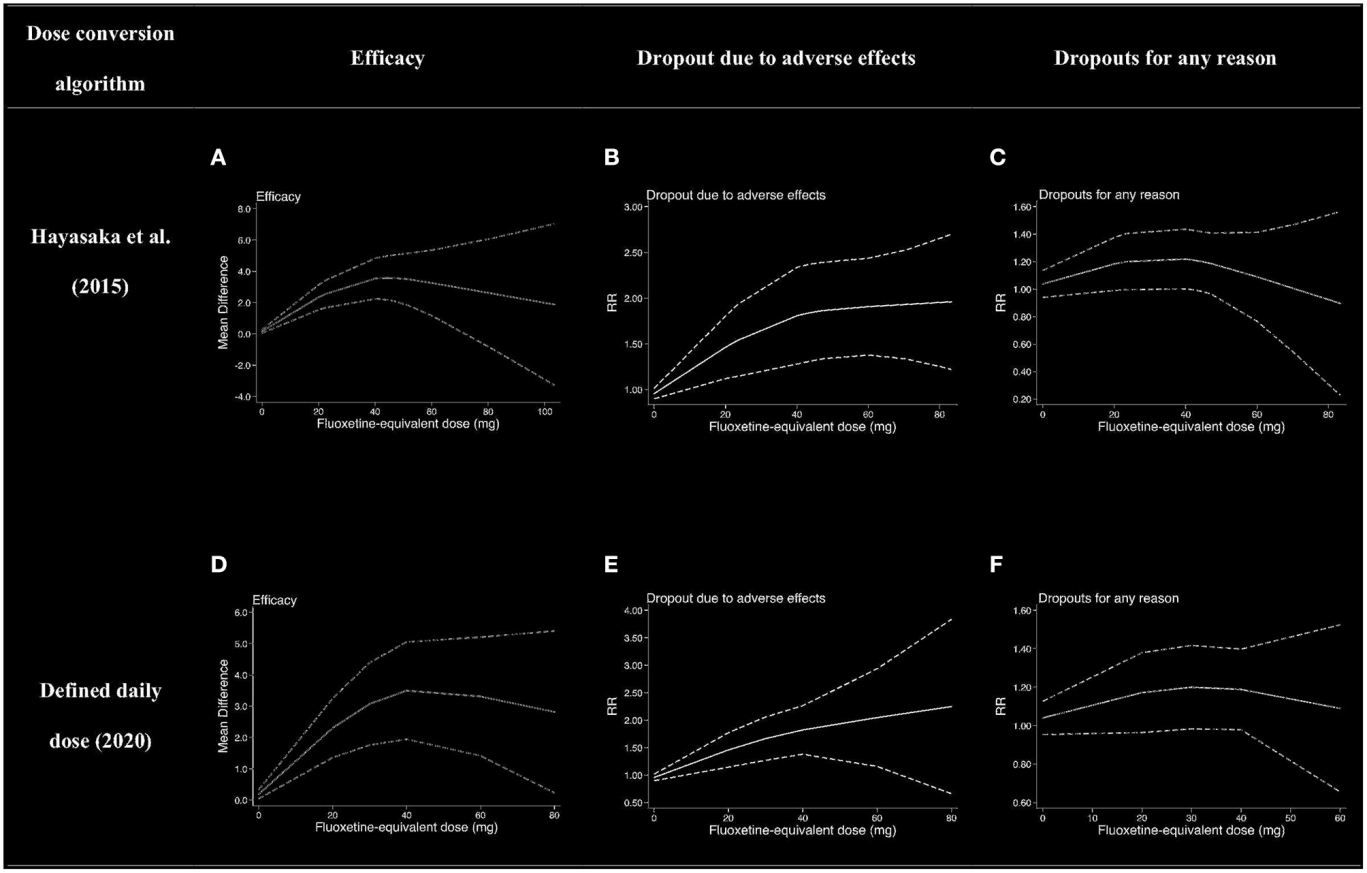
Dose–outcome relationships for serotonin reuptake inhibitors (SRIs) in two dose conversion algorithms. RR, risk ratio. The dotted lines represent 95% CIs. **(A–C)** were the dose–outcome plots using the conversion method of Hayasaka et al. (31). **(D–F)** were the dose–outcome plots using the conversion method of defined daily dose. **(A)** Dose–efficacy relationship for SRIs. **(B)** Dose dropout due to adverse effects relationships for SRIs. **(C)** Dose dropout from all causes of relationships for SRIs. **(D)** Dose–efficacy relationships for SRIs. **(E)** Dose dropout due to adverse effects relationships for SRIs. **(F)** Dose–dropout from all causes relationships for SRIs.

**Figure 5:**
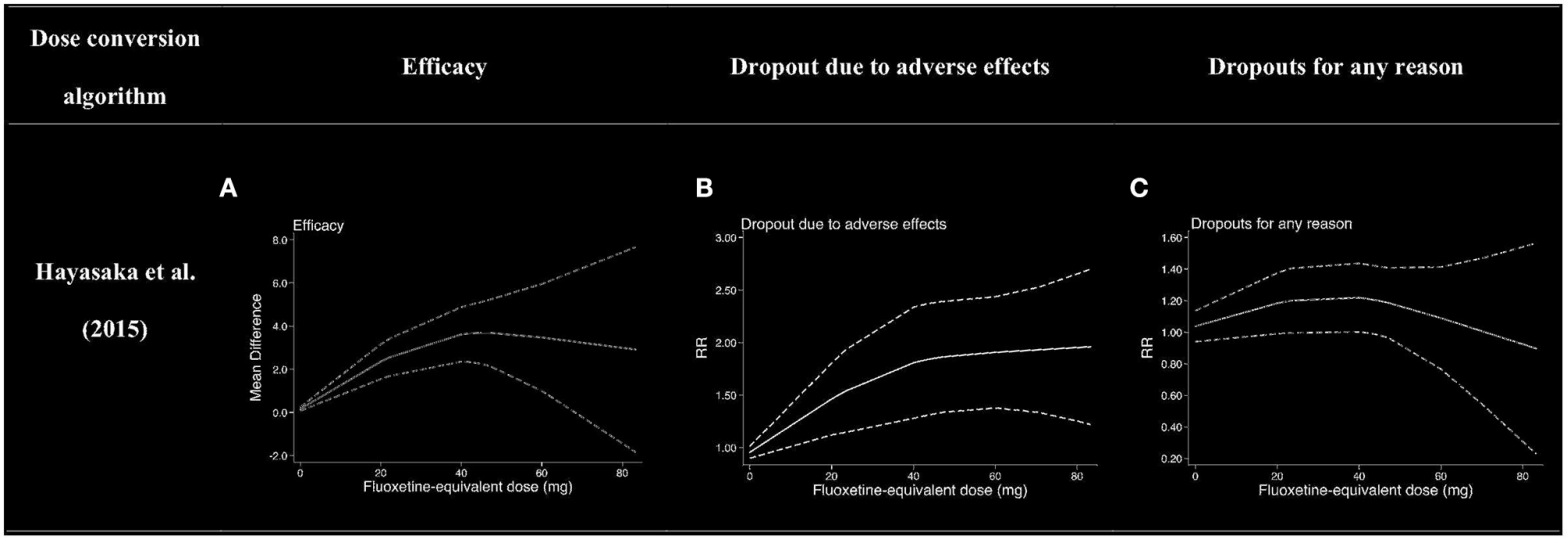
Dose–outcome relationships for selective serotonin reuptake inhibitors (SSRIs). The studies for clomipramine were removed, and the rest were all for SSRIs. The dotted lines represent 95% confidence intervals. **(A)** Dose–efficacy relationship for SRIs. **(B)** Dose dropout due to adverse effects relationships for SRIs. **(C)** Dose dropout from all causes relationships for SRIs.

Due to lack of partial data, nine, seven, and six literatures were included into the dose efficacy and dose dropout due to adverse effects and the dose dropout for all-cause analyses, respectively. Thus, the dose range was slightly different in the analysis. Table 2 displays the suggested start dose, usual maximum doses, and maximum doses occasionally prescribed for each SRI in OCD according to the American Psychiatric Association (APA) dose recommendations (7). The dose–response relationships for SRIs after dose equivalent conversion are presented in Figure 4. Furthermore, we removed the studies of clomipramine and analyzed the dose–outcome relationships for SSRIs and the results are presented in Figure 5.

**Table 2:** Dosing of serotonin reuptake inhibitors in OCD.

**SRI**	**Minimum (mg/day)**	**Maximum (mg/day)**	**Occasionally prescribed maximum dose (mg/day)^a^**
Citalopram	20	80	120
Clomipramine	25	250	^b^
Escitalopram	10	40	60
Fluoxetine	20	80	120
Fluvoxamine	50	300	400
Paroxetine	20	60	100
Sertraline	50	200	400

a *These doses are sometimes used for patients who were rapid metabolizers or with no/mild side effects and inadequate therapeutic response after 8 weeks or more at the usual maximum dose*.

b *Combined plasma levels of clomipramine plus desmethylclomipramine 12 h after the dose should be kept below 500 ng/ml to minimize risk of seizures and cardiac conduction delay*.

### Efficacy

We used MD measured by the Y-BOCS as an index of efficacy. It can be seen from the dose–efficacy curves (Figures 4A,D) that the best efficacy was achieved when the equivalent dose of fluoxetine was about 40 mg. Thus, it can be concluded that within the dose range of 0–100 mg fluoxetine equivalent, about 40 mg was the optimal dose of SRIs for efficacy. Considering the efficacy result of SRIs vs. placebo, no significant heterogeneity was observed in the aggregated mean change of Y-BOCS. The pooled mean change in the SRI-treated group for the Y-BOCS was significantly greater than that in the placebo treatment group, with MD of −3.67 (95% CI, −4.67, −2.68; *I*^2^ = 21%) (Appendix 2). Due to limited evidence, we could not estimate the dose efficacy of individual SRI in the treatment of adult OCD.

### Tolerability

We used dropouts due to adverse effects as an indicator of tolerability. The dose dropout due to adverse-effect curves (Figures 4B,E) showed that RR gradually increased in the dose range of 0–83.7 mg, from 0.95 (95% CI, 0.90, 1.01) for placebo, to 1.46 (95% CI, 1.12–1.80) for 20 mg, 1.81 (95% CI, 1.28–2.34) for 40 mg, 1.91 (95% CI, 1.38–2.43) for 60 mg, and 1.96 (95% CI, 1.22–2.70) for 83.7 mg of fluoxetine equivalent. The relationship between dose and discontinuations due to adverse effects indicates that tolerability decreased with increasing doses within the measured dose range. The point estimates and their 95% CIs of RRs for dropouts due to adverse effects at 0–83.7 mg of fluoxetine equivalents of SRIs are presented in Table 3. There were significant differences in SRI and placebo treatment for adverse events, with RR of 1.77 (95% CI, 1.38–2.28; *I*^2^ = 0%) (Appendix 2).

**Table 3:** RRs for tolerability and acceptability at various doses of SRIs.

**Dose of SRIs (mg)**	**Dropouts due to adverse effects (RR, 95% CI)**	**Dropouts for any reason (RR, 95% CI)**
0.0	0.95 (0.90, 1.01)	1.04 (0.94, 1,14)
20.0	1.46 (1.12, 1.80)	1.18 (0.99, 1.38)
20.3	1.47 (1.12, 1.82)	NR
22.2	1.51 (1.14, 1.89)	1.19 (0.99, 1.39)
23.5	1.54 (1.15, 1.93)	1.20 (1.00, 1.40)
40.0	1.81 (1.28, 2.34)	1.22 (1.00, 1.44)
40.6	1.81 (1.28, 2.34)	NR
41.9	1.83 (1.29, 2.36)	1.21 (1.00, 1.43)
44.4	1.85 (1.31, 2.38)	1.20 (0.98, 1.42)
47.1	1.86 (1.33, 2.39)	1.19 (0.96, 1.41)
60.0	1.91 (1.38, 2.43)	1.09 (0.77, 1.41)
70.6	1.91 (1.38, 2.43)	1.00 (0.53, 1.47)
81.1	1.95 (1.25, 2.66)	NR
83.7	1.96 (1.22, 2.70)	0.89 (0.22, 1.56)

*RRs, relative risks; SRIs, serotonin reuptake inhibitors; CI, confidence interval; NR, none reported. We used dropout due to adverse effects as an index of tolerability and dropouts for any reason as an index of acceptability*.

### Acceptability

The dose–outcome curves (Figures 4C,F) showed that in terms of proportion of dropouts due to any reason, there was no significant tendency. The RR for dropout due to all causes had no significant differences in SRI and placebo treatment. The RR was 1.04 (95% CI, 0.94, 1.14) for placebo, 1.18 (95% CI, 0.99–1·38) for 20 mg, 1.22 (95% CI, 1.00–1.44) for 40 mg, 1.09 (95% CI, 0.77–1.41) for 60 mg, and 0.89 (95% CI, 0.22–1.56) for 83.7 mg of fluoxetine equivalent. The point estimates and their 95% CIs of RRs for dropouts from all causes at 0–83.7 mg of fluoxetine equivalents of SRIs are presented in Table 3. The different SRI dose categories did not differ from placebo or any other in all-cause dropout rates, with a total RR of 1.04 (95% CI, 0.90–1.20; *I*^2^ = 32%) (Appendix 2).

Various knots were examined when we plotted splines for the dose–outcome curves of SRIs (Appendix 2), and all the curves overlapped with our primary analyses. When we examined different dose-equivalence calculations by the method of SRI-defined daily dose, all results were similar to the primary results (Figure 4). To explain the residual heterogeneity, we conducted sensitivity analysis by removing the studies on treatment of clomipramine to test the influence of clomipramine. The results were also consistent with the results of SRIs (Figure 5).

## Discussion

Due to rare evidence of the optimal doses of SRIs for OCD, we conducted a systematic analysis to provide conclusive proof. This meta-analysis was the largest (11 RCTs with 2,322 patients) one to investigate the optimal doses of SRIs for OCD. For SRIs, the efficacy increased at the doses from 0 to 40 mg of fluoxetine equivalents, while it did not increase further or even decrease slightly at doses up to 100 mg. Dropouts due to adverse effects showed a gradual increase in the dose range of 0–83.7 mg. However, dropouts due to any reason were not significantly related to the doses of SRIs. These outcomes suggested that the increased burden of side effects and the stagnation of therapeutic efficacy increase limited the use of higher doses of SRIs in OCD.

Data from our study showed that the effectiveness of SRIs was optimal at the fluoxetine equivalent dose of about 40 mg, and the efficacy decreased as the dose increased higher than 40 mg. The result was partially inconsistent with a previous study on SRIs. Using the dose equivalent conversion method of Bollini et al. (32), Bloch et al. (17) divided the SSRI dose into low, medium, and high and observed that there was a stepwise increase in efficacy with dose and all-cause dropouts were not significantly related to SSRI dose. The possible reasons for the difference between decreased efficacy at higher doses (>40 mg) and other mainstream recommendations for higher initial doses of SRIs for OCD (14, 16) were as follows. First, our study used different dose conversion methods, which might lead to different results. Therefore, in order to improve the reliability of the experimental results, the most comprehensive and the latest dose equivalent conversion methods (31, 34) were used in this study. Second, compared with other studies, the dose classification method we used was different. In our study, the dose was treated as a continuous variable and the spline curve model was used for dose–response analysis, so that it is easier to find the turning point. Third, limited studies included in the study, especially those with a higher dose, may result in bias of results. Fourth, all the studies included in this study were fixed-dose studies, and participants with a large initial dose had increased side effects. This may affect the efficacy results, thereby resulting in bias of the dose-efficacy outcome. Last but not least, the short follow-up time (8–13 weeks) included in this study may also have some influence on the outcomes.

The result of dropouts due to side effects was consistent with other studies; that is, side effects increased with increasing licensed dose. Although the result of all-cause dropouts was consistent with that of a previous study, causes may differ, since the CI of all RR values for dose–all-cause dropout outcome contains 1, indicating that the dose and dropouts due to all causes had no obvious relationship. Bloch et al. (17) concluded that the efficacy and side effects canceled each other out, so there was no significant correlation between all-cause dropouts and dose.

Although both clomipramine and SSRI belong to SRI, their mechanisms of action are different. Clomipramine is a tricyclic antidepressant, which works by inhibiting the reuptake of norepinephrine (NA) and serotonin in the presynaptic membrane. According to previous evidence, the treatment efficacy of clomipramine for OCD is related to its relatively high potency in affecting serotonergic neurotransmission (37). In order to exclude the influence of clomipramine on the research results, clomipramine was removed and analyzed again (Figure 5). The results were found to be completely consistent with the previous results, indicating that clomipramine had no influence on the outcomes. However, there are few studies on clomipramine, which also resulted in certain limitations. More studies are needed to make such conclusion more reliable. In addition, in order to verify the robustness of the results, we set different dose knots and adopted two different dose conversion methods for analysis, and the analysis results were in good agreement.

There were some limitations to this meta-analysis. First, the best method to calculate the dose equivalency among antidepressants was not clear. In our study, we adopted the most comprehensive and latest empirically derived conversion algorithms (31, 34) and examined results through sensitivity analyses, in which different conversion algorithms and multiple knots with different doses were applied. Still, it was difficult to avoid bias caused by the conversion pattern. Second, although we searched a lot of databases, there were too few eligible studies observed and available in our meta-analysis to address the dose–response differences between individual SRIs. Therefore, we meta-analyzed SRIs as a whole, because they were all efficacious and shared a key therapeutic mechanism. Third, all studies included in this meta-analysis shared a relatively similar treatment duration of 8–13 weeks, but there were too few trials to examine treatment duration, which influences SRI efficacy. Fourth, fixed-dose regimens may be considered to not reflect clinical practice, especially when rapid titration regimens are not used or used, and discontinuation may be overestimated due to side effects. However, only through a fixed-dose study can the dose dependence be strictly checked. Fifth, although we obtained a lot of literatures through searching, no fixed-dose RCTs conforming to this study have been conducted in the past 10 years, so all studies included in this paper were relatively old. In addition, findings related to the outcomes of SRIs were based on a small number of participants (*n* = 2,232). Finally, although the funnel plots showed no publication bias, we cannot exclude the possibility of reporting bias because we only included published studies, and the outcomes were not reported in all studies.

There are also various highlights in our study. First of all, we included the most advanced dose–response meta-analysis and regarded dose as a continuous variable, so we could better resolve the point of change and avoid misleading dose classification. In addition, we checked not only the dose dependence of the efficacy but also the tolerability and acceptability. Furthermore, we conducted the study based on the largest and most comprehensive fixed-dose, single-blind, or double-blind RCTs of SRIs in the acute-phase treatment of OCD and took not only SSRIs but also a tricyclic antidepressant, clomipramine, into consideration.

## Conclusion

In conclusion, our analyses showed that the optimal dose of efficacy was reached at about 40 mg fluoxetine equivalent and tolerability decreased with increased doses within the dose range reviewed, but the overall acceptability of treatments appears to be dose-independent. Therefore, we conclude that for most of the patients receiving an SRI for the acute-phase treatment of OCD, the optimal dose should be achieved based on a balance between efficacy and tolerability. Further large-scale prospective researches are needed to rigorously make clearer the utility of higher doses of SRIs in the treatment of OCD and examine the dose–response relationship in specific populations, such as old or pediatric patients. It is noteworthy that there are few specific fixed-dose studies published concerning children and adolescents with OCD, and it is hoped that researchers will pay attention to this issue and conduct related studies in the future.

## Data Availability Statement

The raw data supporting the conclusions of this article will be made available by the authors, without undue reservation.

## Author Contributions

MX and QW conceptualized and designed the study. JX, QH, and RQ conducted the literature search and summary evaluated the papers for inclusion and exclusion criteria. MX, JX, QH, and MD conducted all statistical analyses and hammered away at methodology. All authors contributed to the interpretation of the manuscript and have approved the final manuscript.

## Funding

This study was financially supported by the National Natural Science Foundation of China (Grant No. 81771446), the National Key Research and Development Program of China (Grant No. 2018YFC1314300). This study was also supported by the Ministry of Science and Technology of China (Grant No. 2016YFC1307003).

## Conflict of Interest

The authors declare that the research was conducted in the absence of any commercial or financial relationships that could be construed as a potential conflict of interest.

## Publisher's Note

All claims expressed in this article are solely those of the authors and do not necessarily represent those of their affiliated organizations, or those of the publisher, the editors and the reviewers. Any product that may be evaluated in this article, or claim that may be made by its manufacturer, is not guaranteed or endorsed by the publisher.
