# Supplementary material for: Optimal Dose of Serotonin Reuptake Inhibitors for Obsessive-Compulsive Disorder in Adults: A Systematic Review and Dose–Response Meta-Analysis
Source: Front Psychiatry. 2021 Sep 23;12:717999. doi: 10.3389/fpsyt.2021.717999 (PMC8495022; doi:10.3389/fpsyt.2021.717999)
Supplement: Supplementary file 1 [file Data_Sheet_1.pdf]

Supplementary Material

1 Appendix 1: Search strategy

Appendix 1: Search strategy
Database: Ovid MEDLINE(R) and Epub Ahead of Print, In-Process & Other Non-Indexed Citations, Daily and Versions(R) <1946 to Present> 22-02-20
1. Obsessive-Compulsive Disorder/
2. (obses* adj2 compuls*).ti,ab.
3. (ocd or obsessivecompuls* or anankast* or anancast*).ti,ab.
4. 1 or 2 or 3
5. (antidepress* or anti-depress* or ssri* or sri or sris or selective serotonin reuptake inhibitor* or reuptake inhibitor* or re-uptake inhibitor* or 5-HT Uptake Inhibitor* or psychotropic* or TCAs or Tricyclic Antidepressant*).ti,ab.
6. (citalopram* or escitalopram* or fluoxetin* or fluvoxamin* or paroxetin* or sertralin* or clomipramin*).ti,ab,rn.
7. 5 or 6
8. randomized controlled trial.pt.
9. controlled clinical trial.pt.
10. randomized.ti,ab.
11. placebo.ti,ab.
12. clinical trials.ti,ab.
13. randomly.ti,ab.
14. trial.ti,ab.
15. 8 or 9 or 10 or 11 or 12 or 13 or 14
16. 4 and 7 and 15

Database: Embase 1974 to 2020 22-02-20
1. Obsessive-Compulsive Disorder/
2. (obses* adj2 compuls*).ti,ab.
3. (ocd or obsessivecompuls* or anankast* or anancast*).ti,ab.
4. 1 or 2 or 3
5. (antidepress* or anti-depress* or ssri* or sri or sris or selective serotonin reuptake inhibitor* or reuptake inhibitor* or re-uptake inhibitor* or 5-HT Uptake Inhibitor* or psychotropic* or TCAs or Tricyclic Antidepressant*).ti,ab.
6. (citalopram* or escitalopram* or fluoxetin* or fluvoxamin* or paroxetin* or sertralin* or clomipramin*).ti,ab,rn.
7. 5 or 6
8. randomized.ti,ab.
9. placebo.ti,ab.
10. clinical trials.ti,ab.
11. randomly.ti,ab.
12. trial.ti,ab.
13. exp randomized controlled trial/
14. exp controlled clinical trial/
15. 8 or 9 or 10 or 11 or 12 or 13 or 14

16. 4 and 7 and 15

Database: BIOSIS Previews 1995 to 2020 22-02-20

1. Obsessive-Compulsive Disorder.mp.
2. (obses* adj2 compuls*).ti,ab.
3. (ocd or obsessivecompuls* or anankast* or anancast*).ti,ab.
4. 1 or 2 or 3
5. (antidepress* or anti-depress* or ssri* or sri or sris or selective serotonin reuptake inhibitor* or reuptake inhibitor* or re-uptake inhibitor* or 5-HT Uptake Inhibitor* or psychotropic* or TCAs or Tricyclic Antidepressant*).ti,ab.
6. (citalopram* or escitalopram* or fluoxetin* or fluvoxamin* or paroxetin* or sertralin* or clomipramin*).ti,ab,rn.
7. 5 or 6
8. randomized controlled trial.mp.
9. controlled clinical trial.mp.
10. randomized.ti,ab.
11. placebo.ti,ab.
12. clinical trials.ti,ab.
13. randomly.ti,ab.
14. trial.ti,ab.
15. 8 or 9 or 10 or 11 or 12 or 13 or 14
16. 4 and 7 and 15

Database: PsycINFO 22-02-20

1. Obsessive Compulsive Disorder.mp.
2. (obses* adj2 compuls*).ti,ab.
3. (ocd or obsessivecompuls* or anankast* or anancast*).ti,ab.
4. 1 or 2 or 3
5. (antidepress* or anti-depress* or ssri* or sri or sris or selective serotonin reuptake inhibitor* or reuptake inhibitor* or re-uptake inhibitor* or 5-HT Uptake Inhibitor* or psychotropic* or TCAs or Tricyclic Antidepressant*).ti,ab.
6. (citalopram* or escitalopram* or fluoxetin* or fluvoxamin* or paroxetin* or sertralin* or clomipramin*).ti,ab.
7. 5 or 6
8. randomized controlled trial.af.
9. controlled clinical trial.af.
10. randomized.ti,ab.
11. placebo.ti,ab.
12. clinical trials.ti,ab.
13. randomly.ti,ab.
14. trial.ti,ab.
15. 8 or 9 or 10 or 11 or 12 or 13 or 14
16. 4 and 7 and 15

Database: Cochrane Central Register of Controlled Trials (CENTRAL) 22-02-20

("Obsessive Compulsive Disorder" or (obses* NEAR compuls*) or (ocd or obsessivecompuls* or anankast* or anancast*) in Title Abstract Keyword) AND ((antidepress* or anti-depress* or ssri* or sri or sris or "selective serotonin reuptake inhibitor*" or "reuptake inhibitor*" or "re-uptake inhibitor*" or "5-HT Uptake Inhibitor*" or psychotropic* or TCAs or Tricyclic Antidepressant*) or (citalopram* or escitalopram* or fluoxetin* or fluvoxamin* or paroxetin* or sertralin* or

clomipramin*) in Title Abstract Keyword) AND ("randomized controlled trial" OR "controlled clinical trial" OR randomized OR placebo OR "clinical trials" OR randomly OR trial NOT (animals NOT humans)) in Title Abstract Keyword (Word variations have been searched) Last
saved: 22/02/2020 08:44(8)

Database: Web of science 22-02-20

9. #8 AND #7 AND #4
8. TS=(randomized controlled trial OR controlled clinical trial OR randomized OR placebo OR clinical trials OR randomly OR trial NOT (animals NOT humans))
7. #6 OR #5
6. TS=(citalopram* or escitalopram* or fluoxetin* or fluvoxamin* or paroxetin* or sertralin* or clomipramin*)
5. TS=(antidepress* or anti-depress* or ssri* or sri or sris or selective serotonin reuptake inhibitor* or reuptake inhibitor* or re-uptake inhibitor* or 5-HT Uptake Inhibitor* or psychotropic* or TCAs or Tricyclic Antidepressant*)
4. #3 OR #2 OR #1
3. TS=(ocd or obsessivecompuls* or anankast* or anancast*)
2. TS=(obses* NEAR compuls*)
1. TS=(Obsessive Compulsive Disorder)

Database CINAHL 22-02-20

S1 TX Obsessive Compulsive Disorder OR TX (obses* adj2 compuls*) OR TX ((ocd or obsessivecompuls* or anankast* or anancast*))
S2 TX (antidepress* or anti-depress* or ssri* or sri or sris or selective serotonin reuptake inhibitor* or reuptake inhibitor* or re-uptake inhibitor* or 5-HT Uptake Inhibitor* or psychotropic* or TCAs or Tricyclic Antidepressant*) OR TX (citalopram* or escitalopram* or fluoxetin* or fluvoxamin* or paroxetin* or sertralin* or clomipramin*)
S3 PT randomized controlled trial OR TX (controlled clinical trial OR randomized OR placebo OR clinical trials OR randomly OR trial) NOT TX (animals NOT humans)
S4 S1 AND S2 AND S3

2 Appendix 2: Supplementary Figures

2.1 Forest and funnel plots

	SRIs			placebo				Mean Difference
Study or Subgroup	Mean	SD	Total	Mean	SD	Total	Weight	IV, Fixed, 95% CI
Jenike 1990 Ser	−2.2	8.09	10	−0.5	6.81	9	2.2%	−1.70 [−8.40, 5.00]
Montgomery 1993 Fluo	−5.32	6.74	53	−3.7	5.98	57	17.5%	−1.62 [−4.01, 0.77]
Montgomery 2001 Cit	−9.23	7.07	100	−5.6	6.9	101	26.7%	−3.63 [−5.56, −1.70]
Nakajima 1996 Fluv	−7.2	7.05	31	−1.9	7.2	33	8.2%	−5.30 [−8.79, −1.81]
Stein 2007 Esc	−11.75	8.32	114	−8.46	8.11	114	21.9%	−3.29 [−5.42, −1.16]
Tollefson 1994 Fluo	−5.92	7.16	88	−0.7	6.78	89	23.6%	−5.22 [−7.27, −3.17]
**Total (95% CI)**			**396**			**403**	**100.0%**	**−3.67 [−4.67, −2.68]**

Heterogeneity: $Chi^2 = 6.31$, df = 5 (P = 0.28); $I^2 = 21\%$
Test for overall effect: Z = 7.21 (P < 0.00001)

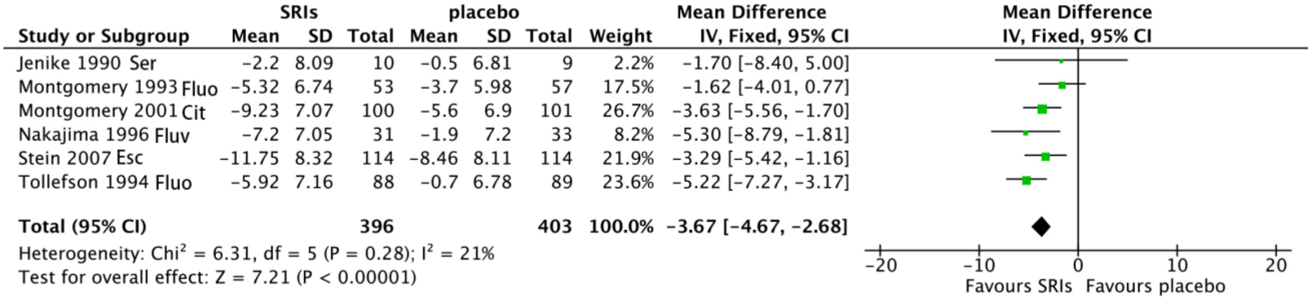

Supplementary Figure 1. The forest plot of comparison of the mean change from baseline of Y-BOCS (95% confidence interval) of SRIs versus placebo in OCD adults. CI, confidence interval; Y-BOCS, Yale-Brown Obsessive Compulsive Scale; IV, inverse variance; df, degrees of freedom; SD, standard deviation; OCD, obsessive-compulsive disorder; SRIs, serotonin re-uptake inhibitors. Ser, Sertraline; Fluo, Fluoxetine; Cit, Citalopram; Fluv, Fluvoxamine; Esc, escitalopram.

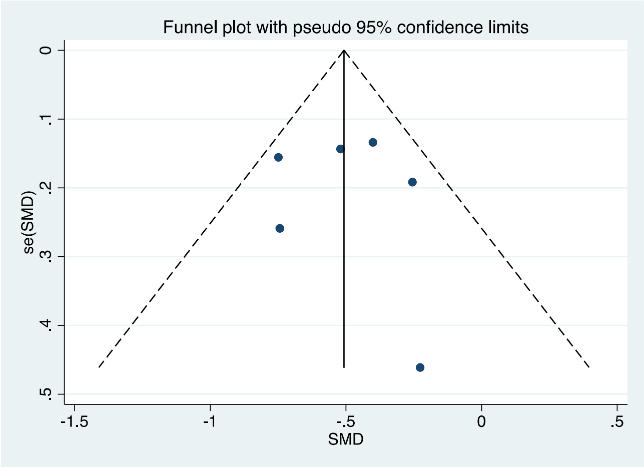

Supplementary Figure 2. The funnel plot to determine the reporting bias of the studies which comparison of the mean change from baseline of Y-BOCS (95% confidence interval) of SRIs versus placebo in OCD adults. Y-BOCS, Yale-Brown Obsessive Compulsive Scale; OCD, obsessive-compulsive disorder; SRIs, serotonin re-uptake inhibitors.

Study or Subgroup	SRIs Events	SRIs Total	placebo Events	placebo Total	Weight	Risk Ratio M-H, Fixed, 95% CI
Greist 1995 Ser 20.3	6	80	5	84	5.6%	1.26 [0.40, 3.97]
Greist 1995 Ser 40.6	11	81	5	84	5.6%	2.28 [0.83, 6.28]
Greist 1995 Ser 81.1	6	80	5	84	5.6%	1.26 [0.40, 3.97]
Hollander 2003 Par 23.5	9	88	7	89	7.9%	1.30 [0.51, 3.34]
Hollander 2003 Par 47.1	8	86	7	89	7.8%	1.18 [0.45, 3.12]
Hollander 2003 Par70.6	12	85	7	89	7.8%	1.79 [0.74, 4.34]
Montgomery 1993 Fluo 20	4	52	2	56	2.2%	2.15 [0.41, 11.27]
Montgomery 1993 Fluo 40	2	52	2	56	2.2%	1.08 [0.16, 7.37]
Montgomery 1993 Fluo 60	1	54	2	56	2.2%	0.52 [0.05, 5.55]
Montgomery 2001 Cit 20	4	102	2	101	2.3%	1.98 [0.37, 10.57]
Montgomery 2001 Cit 40	6	98	2	101	2.2%	3.09 [0.64, 14.95]
Montgomery 2001 Cit 60	4	100	2	101	2.3%	2.02 [0.38, 10.78]
Nakajima 1996 Fluv 41.7	2	34	4	33	4.6%	0.49 [0.10, 2.47]
Nakajima 1996 Fluv 83.3	6	27	4	33	4.1%	1.83 [0.58, 5.84]
Stein 2007 Cit 22.2	10	113	9	114	10.2%	1.12 [0.47, 2.65]
Stein 2007 Cit 44.4	13	114	9	114	10.2%	1.44 [0.64, 3.24]
Stein 2007 Par 47.1	18	117	9	114	10.4%	1.95 [0.91, 4.16]
Tollefson 1994 Fluo 20	3	87	2	89	2.3%	1.53 [0.26, 8.96]
Tollefson 1994 Fluo 40	12	89	2	89	2.3%	6.00 [1.38, 26.04]
Tollefson 1994 Fluo 60	17	90	2	89	2.3%	8.41 [2.00, 35.32]
**Total (95% CI)**		**1629**		**1665**	**100.0%**	**1.77 [1.38, 2.28]**
Total events	154		89			

Heterogeneity: $Chi^2 = 14.88$, df = 19 (P = 0.73); $I^2 = 0\%$
Test for overall effect: Z = 4.46 (P < 0.00001)

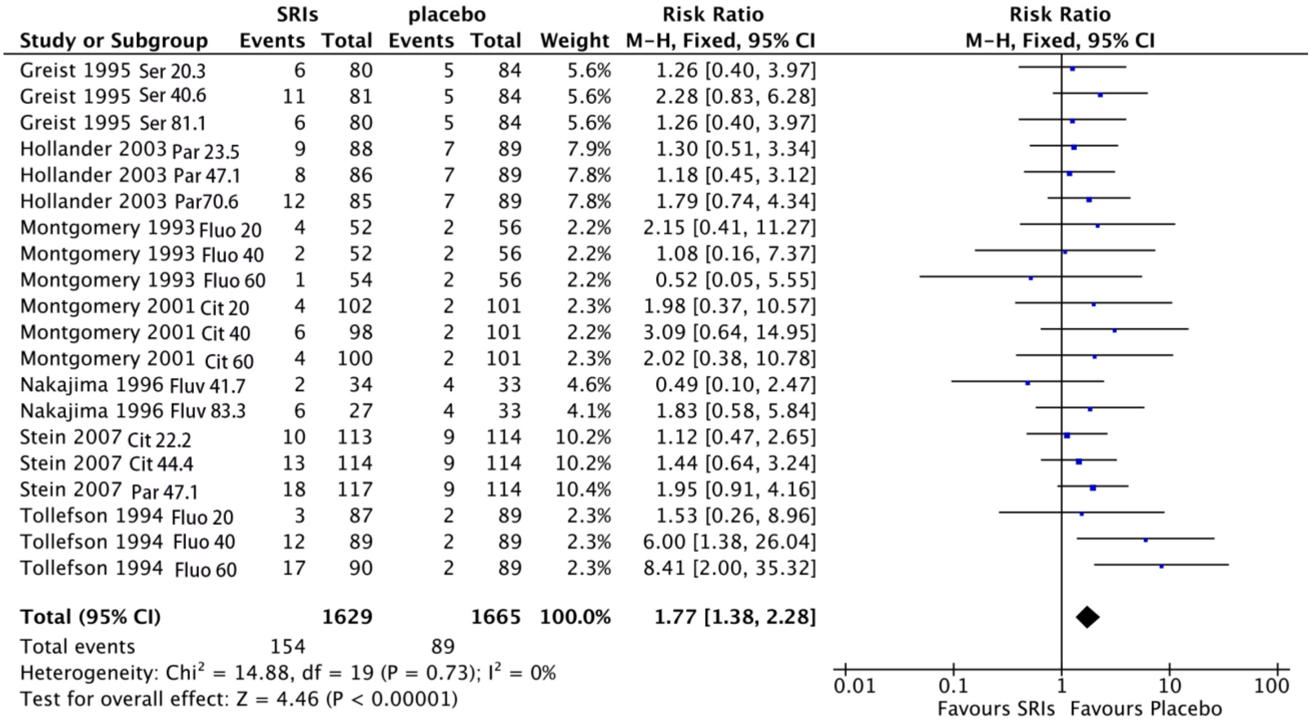

Supplementary Figure 3. The forest plot of comparison of relative risk (95% confidence interval) for dropout due to side effects of SRIs versus placebo in OCD adults. CI, confidence interval; M-H,Mantel-Haenszel; df, degrees of freedom; OCD, obsessive-compulsive disorder; SRIs, serotonin re-uptake inhibitors. Ser, Sertraline; Par, Paroxetine; Fluo, Fluoxetine; Cit, Citalopram; Fluv, Fluvoxamine. The number after the agent represents the dose converted into fluoxetine equivalent, and the unit is mg.

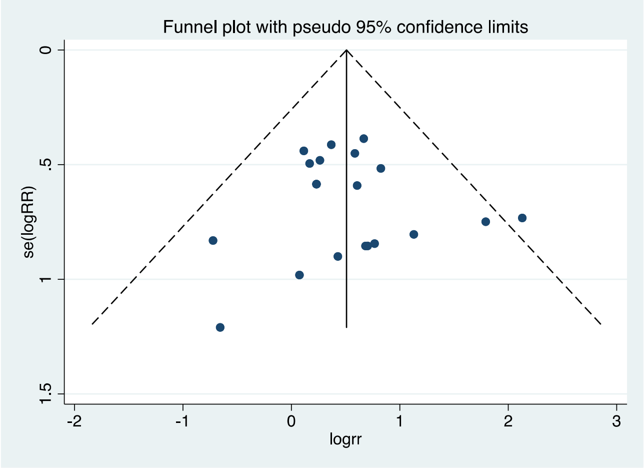

Supplementary Figure 4. The funnel plot to determine the reporting bias of the studies which comparison of relative risk (95% confidence interval) for dropout due to side effect of SRIs versus placebo in OCD adults. Y-BOCS, Yale-Brown Obsessive Compulsive Scale; OCD, obsessive-compulsive disorder; SRIs, serotonin re-uptake inhibitors.

Study or Subgroup	SRIs Events	SRIs Total	Placebo Events	Placebo Total	Weight	Risk Ratio M-H, Fixed, 95% CI
Hollander 2003 ,Par 23.5	14	88	15	89	5.4%	0.94 [0.48, 1.84]
Hollander 2003 Par 47.1	20	86	15	89	5.4%	1.38 [0.76, 2.51]
Hollander 2003 Par 70.6	19	85	15	89	5.3%	1.33 [0.72, 2.44]
Montgomery 1993 Fluo 20	14	52	15	56	5.3%	1.01 [0.54, 1.87]
Montgomery 1993 Fluo 40	13	52	15	56	5.3%	0.93 [0.49, 1.77]
Montgomery 1993 Fluo 60	14	54	15	56	5.4%	0.97 [0.52, 1.81]
Montgomery 2001 Cit 20	16	102	17	101	6.2%	0.93 [0.50, 1.74]
Montgomery 2001 Cit 40	16	98	17	101	6.1%	0.97 [0.52, 1.81]
Montgomery 2001 Cit 60	15	100	17	101	6.2%	0.89 [0.47, 1.68]
Nakajima 1996 Fluv 41.7	7	34	16	33	5.9%	0.42 [0.20, 0.90]
Nakajima 1996 Fluv 83.3	7	27	16	33	5.2%	0.53 [0.26, 1.11]
Stein 2007 Esc 22.2	37	113	22	114	8.0%	1.70 [1.07, 2.69]
Stein 2007 Esc 44.4	12	114	22	114	8.0%	0.55 [0.28, 1.05]
Stein 2007 Par 47.1	22	117	22	114	8.1%	0.97 [0.57, 1.66]
Tollefson 1994 Fluo 20	12	87	13	89	4.7%	0.94 [0.46, 1.95]
Tollefson 1994 Fluo 40	22	89	13	89	4.7%	1.69 [0.91, 3.14]
Tollefson 1994 Fluo 60	22	90	13	89	4.8%	1.67 [0.90, 3.11]
**Total (95% CI)**		**1388**		**1413**	**100.0%**	**1.04 [0.90, 1.20]**
Total events	282		278			

Heterogeneity: $Chi^2 = 23.70$, df = 16 (P = 0.10); $I^2 = 32\%$
Test for overall effect: Z = 0.49 (P = 0.62)

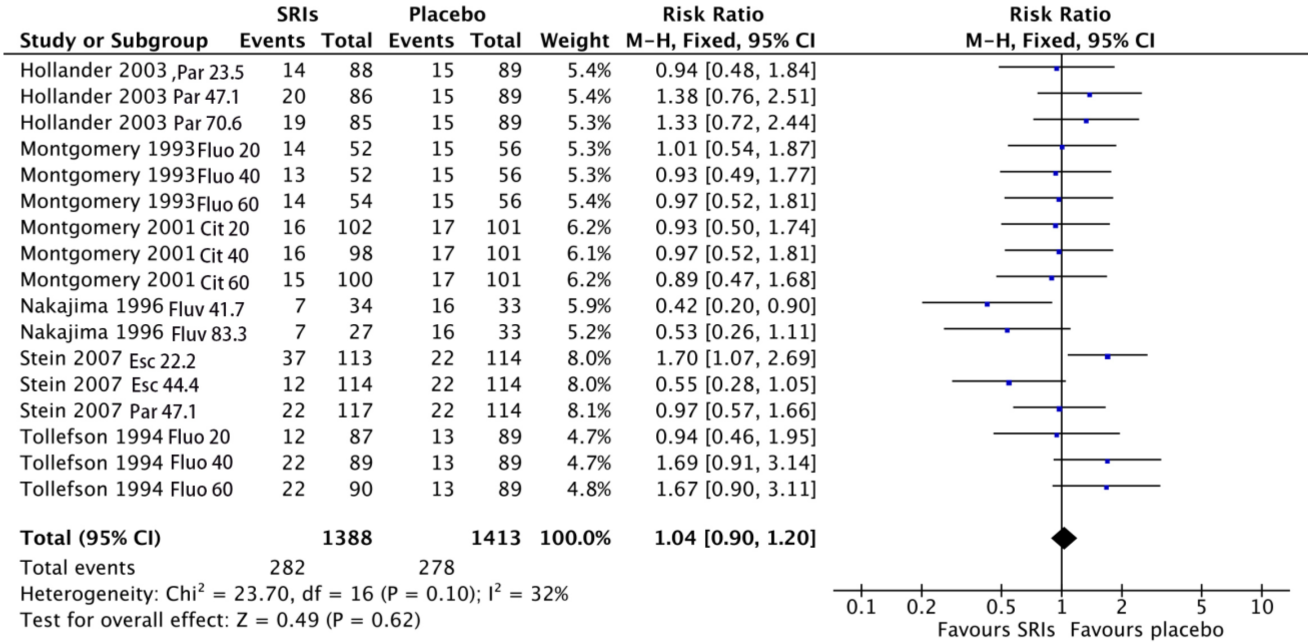

Supplementary Figure 5. The forest plot of comparison of relative risk (95% confidence interval) for dropout for any reason of SRIs versus placebo in OCD adults. CI, confidence interval; M-H,Mantel-Haenszel; df, degrees of freedom; OCD, obsessive-compulsive disorder; SRIs, serotonin re-uptake inhibitors. Par, Paroxetine; Fluo, Fluoxetine; Cit, Citalopram; Fluv, Fluvoxamine; Esc, escitalopram; Par, Paroxetine. The number after the agent represents the dose converted into fluoxetine equivalent, and the unit is mg.

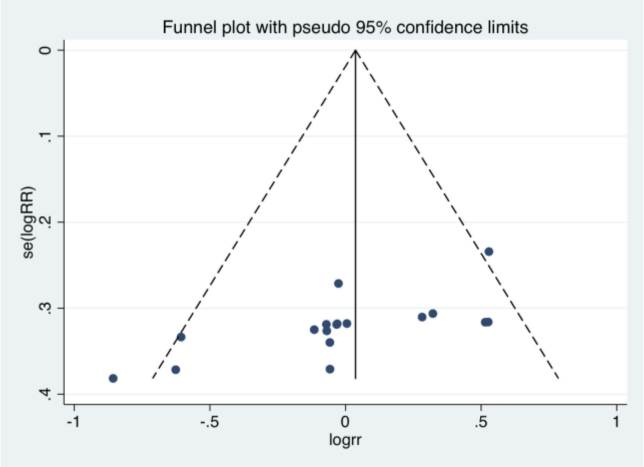

Supplementary Figure 6. The funnel plot to determine the reporting bias of the studies which comparison of relative risk (95% confidence interval) for dropout for any reason of SRIs versus placebo in OCD adults. Y-BOCS, Yale-Brown Obsessive Compulsive Scale; OCD, Obsessive-Compulsive Disorder; SRIs, serotonin re-uptake inhibitors.

2.2 Dose-outcome curves

Knots	Response	Dropout due to adverse effects	Dropouts from all causes
(10 30 50)	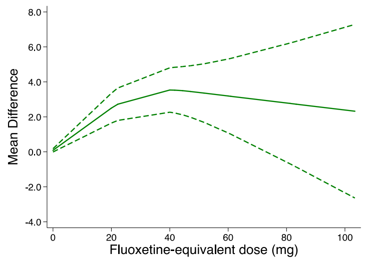	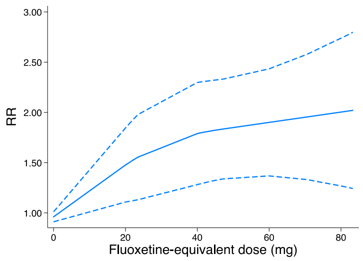	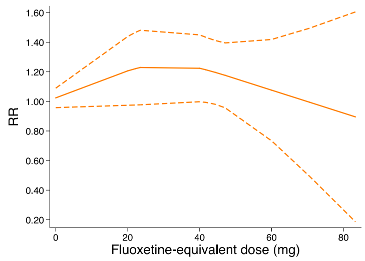
(20 30 40)	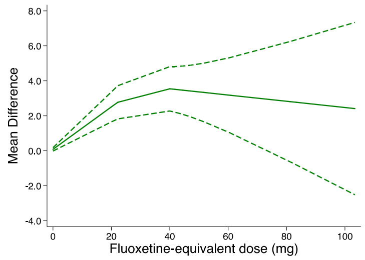	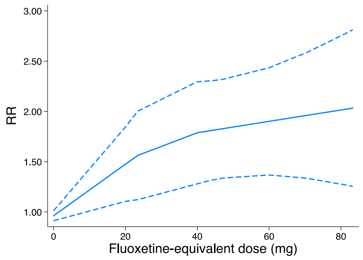	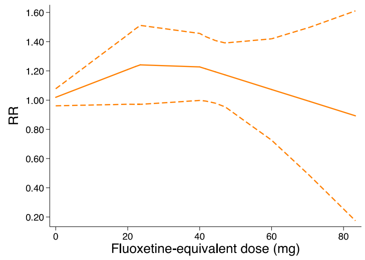
(15 30 45)	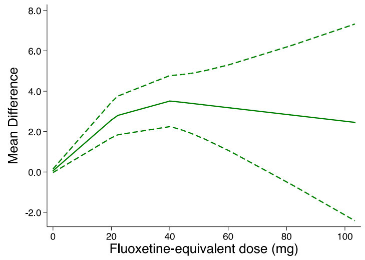	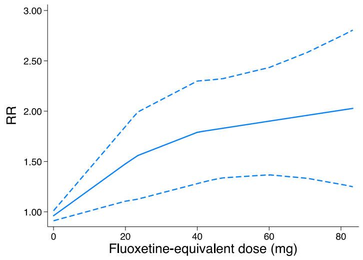	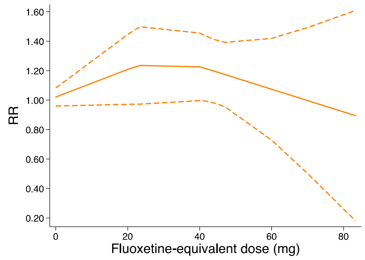
(20 40 60)	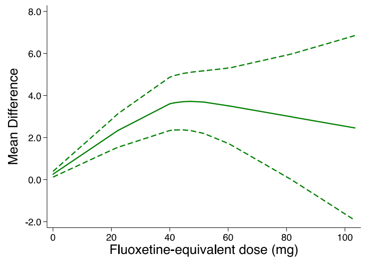	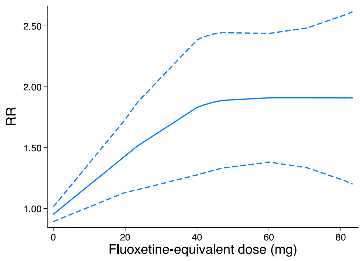	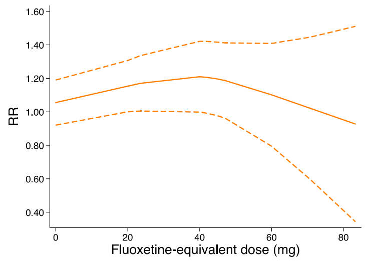

(20 30 50)

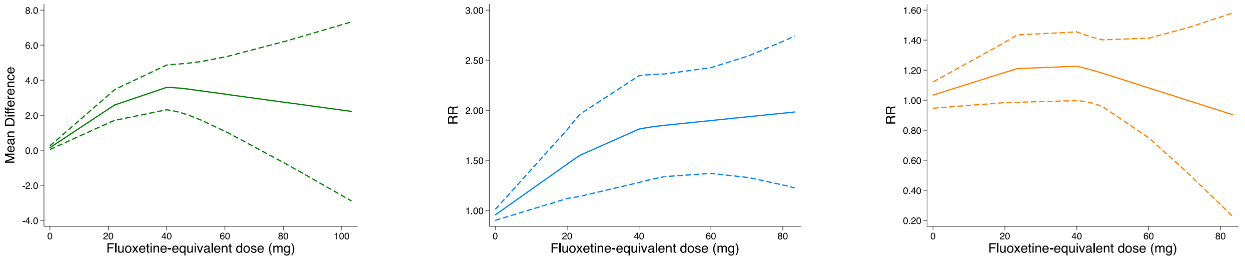

(10 20 40)

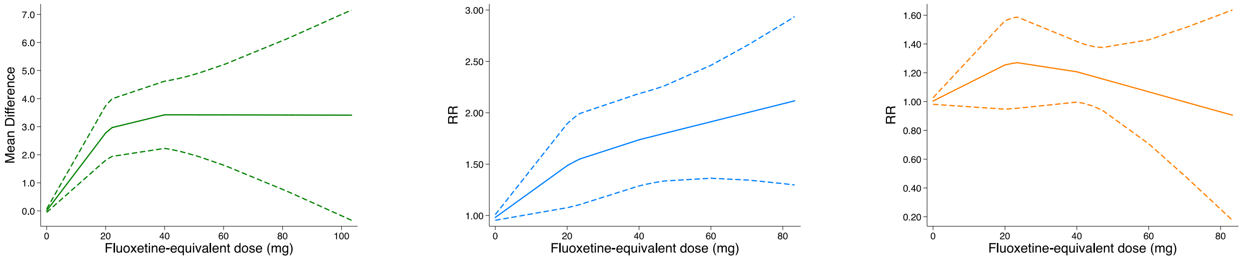

(10 20 30)

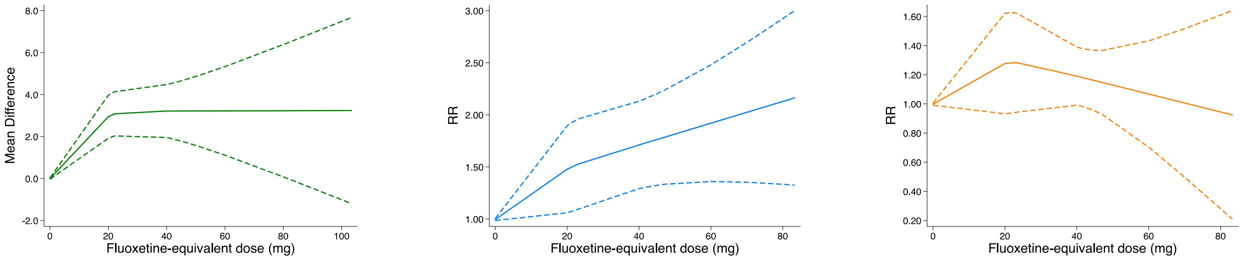

Supplementary Figure 7. Dose-outcome curves of SRIs using various knots. Dose conversion algorithm: Hayasaka et al (2015). The dotted lines represent 95% confidence intervals.

3 Appendix 3: Supplementary Tables

Supplementary table 1. Characteristics of the included studies

Study Name	Drug	No. randomized baseline	Mean dose delivered (mg)	Mean age (SD)	%Female	mean (SD) duration of OCD (years)	Blinding	Dosing Schedule	Baseline Severity Scale	Diagnostic Criteria	Timing of outcome assessment (weeks)	Mean Baseline Severity
(Bogetto et al., 2002)	sertraline (RT)	14	150	NR	NR	NR	1	fixed	Y-BOCS	DSM-IV	12	23.1
	sertraline (ST)	13	150	NR	NR	NR	1	fixed	Y-BOCS	DSM-IV	12	22.9
(Greist et al., 1995)	sertraline	80	50	39.6(13)	55	5.0 (9)	2	fixed	Y-BOCS	DSM-III-R	12	23.2
	sertraline	81	100	40.1 (12)	55	4.7 (7)	2	fixed	Y-BOCS	DSM-III-R	12	24.7
	sertraline	80	200	39.1 (13)	50	5.8 (12)	2	fixed	Y-BOCS	DSM-III-R	12	23.5
	placebo	84	0	35.9 (13)	49	4.6 (8)	2	fixed	Y-BOCS	DSM-III-R	12	23.4
(Hollander et al., 2003)	paroxetine	88	20	40.2 (13.4)	27.3	NR	2	fixed	Y-BOCS	DSM-III-R	12	25.9
	paroxetine	86	40	42.1 (12.7)	27.9	NR	2	fixed	Y-BOCS	DSM-III-R	12	25.4
	paroxetine	85	60	40.0 (15.4)	17.6	NR	2	fixed	Y-BOCS	DSM-III-R	12	25.3
	placebo	89	0	43.1 (12.3)	32.6	NR	2	fixed	Y-BOCS	DSM-III-R	12	25.6
(Jenike et al., 1990)	sertraline	10	200	35.0 (14.0)	20	18 (13)	2	fixed	Y-BOCS	DSM-III	10	22.8
	placebo	9	0	45.0 (13.0)	22.2	22 (11)	2	fixed	Y-BOCS	DSM-III	10	22.8
(López-Ibor et al., 1996)	fluoxetine	30	40	32.4 (10.2)	76.7	NR	2	fixed	Y-BOCS	DSM-III-R	8	27.6
	clomipramine	25	150	35.9 (13.7)	44	NR	2	fixed	Y-BOCS	DSM-III-R	8	25.6
(Milanfranchi et al., 1997)	fluvoxamine	13	300	27.2 (6.8)	30.8	NR	2	fixed	Y-BOCS	DSM-III-R	9	27.5
	clomipramine	13	300	27.5 (5.9)	53.8	NR	2	fixed	Y-BOCS	DSM-III-R	9	29.7
	fluoxetine	52	20	39.11 (11.9)	48.1	NR	2	fixed	Y-BOCS	DSM-III-R	8	23.8

(Montgomery et al., 1993)	fluoxetine	52	40	36.29 (11.9)	51.9	NR	2	fixed	Y-BOCS	DSM-III-R	8	25.5
	fluoxetine	54	60	37.0 (13.0)	46.3	NR	2	fixed	Y-BOCS	DSM-III-R	8	23
	placebo	56	0	36.3 (16.2)	41.1	NR	2	fixed	Y-BOCS	DSM-III-R	8	23.2
(Montgomery et al., 2001)	Citalopram	102	20	37.5 (11.8)	53.9	15.2 (11.7)	2	fixed	Y-BOCS	DSM-IV	12	25.1
	Citalopram	98	40	37.7 (11.1)	54.1	15.4 (10.8)	2	fixed	Y-BOCS	DSM-IV	12	26
	Citalopram	100	60	37.5 (10.9)	58	16.3 (11.6)	2	fixed	Y-BOCS	DSM-IV	12	25.9
	placebo	101	0	38.6 (12.1)	50	16.8 (12.1)	2	fixed	Y-BOCS	DSM-IV	12	25.4
(Nakajima et al., 1996)	fluvoxamine	34	150	30.9 (12.4)	29.4	2.6 (4.1)	2	fixed	JY-BOCS	DSM-III-R	8	24
	fluvoxamine	27	300	36.3 (14.6)	63.3	3.9 (5.7)	2	fixed	JY-BOCS	DSM-III-R	8	25.7
	placebo	33	0	36.4 (15.3)	48.5	4.3 (8.1)	2	fixed	JY-BOCS	DSM-III-R	8	26.2
(Stein et al., 2007)	escitalopram	113	10	38.4 (11.8)	61.1	3.4 (4.4)	2	fixed	Y-BOCS	DSM-IV-TR	12	26.6
	escitalopram	114	20	37.6 (11.7)	57.9	4.8 (6.3)	2	fixed	Y-BOCS	DSM-IV-TR	12	26.6
	paroxetine	117	40	37.4 (11.8)	53.8	4.6 (6.2)	2	fixed	Y-BOCS	DSM-IV-TR	12	27.3
	placebo	114	0	37.6 (11.8)	55.3	3.6 (4.1)	2	fixed	Y-BOCS	DSM-IV-TR	12	27.7
(Tollefson et al., 1994)	fluoxetine	87	20	38.0 (12.7)	52.9	NR	2	fixed	Y-BOCS	DSM-III-R	13	23.6
	fluoxetine	89	40	37.0 (10.8)	55.1	NR	2	fixed	Y-BOCS	DSM-III-R	13	23.5
	fluoxetine	90	60	35.4 (11.3)	61.1	NR	2	fixed	Y-BOCS	DSM-III-R	13	24.4
	placebo	89	0	37.2 (12.3)	51.7	NR	2	fixed	Y-BOCS	DSM-III-R	13	24.3

Abbreviations: SD, standard deviation; OCD, Obsessive-Compulsive Disorder; RT, Rapid Titration; ST, Slow Titration; NR, None Reported; Y-BOCS, Yale–Brown Obsessive Compulsive Scale; JY-BOCS, Japanese Yale–Brown Obsessive Compulsive Scale; DSM-IV, Diagnostic and Statistical Manual of Mental Disorders, Fourth Edition; DSM-III-R, Diagnostic and Statistical Manual of Mental Disorders, Third Edition, Revised; DSM-IV-TR, Diagnostic and Statistical Manual of Mental Disorders, Fourth Edition-Text Revision.
